# TikTok as a source of information for migraine: a content quality analysis

**DOI:** 10.1093/oodh/oqag019

**Published:** 2026-08-04

**Authors:** Isabella M de Jesus, Regor David N Balonga, Liamuel Giancarlo V Untalan, Roland Dominic G Jamora

**Affiliations:** Department of Neurosciences, College of Medicine and Philippine General Hospital, University of the Philippines Manila, Taft Ave., Ermita, Manila, 1000, Philippines; Department of Neurosciences, College of Medicine and Philippine General Hospital, University of the Philippines Manila, Taft Ave., Ermita, Manila, 1000, Philippines; Department of Neurosciences, College of Medicine and Philippine General Hospital, University of the Philippines Manila, Taft Ave., Ermita, Manila, 1000, Philippines; Department of Neurosciences, College of Medicine and Philippine General Hospital, University of the Philippines Manila, Taft Ave., Ermita, Manila, 1000, Philippines; Section of Neurology, Department of Internal Medicine, Cardinal Santos Medical Center, 10 Wilson St., Greenhills West, San Juan City, 1502, Philippines

**Keywords:** migraine, TikTok, social media, health information, quality analysis

## Abstract

Migraine is one of the most common neurological conditions affecting people worldwide. Migraine is the second leading cause of the global burden of neurological diseases. Studies have found the information on TikTok on migraine to be inaccurate, yet it has been a source of health information among the public. The objective of the study was to describe the TikTok videos discussing migraine, to evaluate its content in terms of reliability, quality, and understandability, and investigate the factors of these videos. A search was conducted using the keyword ‘#migraine’ and the top videos were assessed by two independent raters. Video parameters such as type of uploader, views, likes, comments, shares, and duration were also collected. The evaluated videos had low quality and reliability on the Global Quality Scale (GQS) and the modified DISCERN tool, with low understandability and actionability. Higher scores overall in Patient Education Materials Assessment Tool (PEMAT) showed a positive correlation with higher scores on the GQS. Video length (in seconds) had a positive correlation with GQS and PEMAT scores [length with GQS *r* = 0.393 (*P* < .001); length with PEMAT Total *r* = 0.420 (*P* < .001)]. TikTok videos on migraine were found to be of low quality, reliability, and understandability. These findings highlight the role of social media in contributing to the health illiteracy of its audiences, further lobbying that healthcare institutions should create official social media accounts to spread reliable health information to the public on migraines.

## Introduction

Migraine is one of the most common neurological conditions affecting people worldwide [1]. It is a primary headache disorder that is characterized as recurrent, episodic, unilateral headache that ranges from moderate to severe in intensity, accompanied by a pre-drome and/or postdrome [1]. Migraine is the second leading cause of the global burden of neurological diseases, with its prevalence at ~14% of the entire population [1]. A recent study on the global burden of migraine showed that there was an increased prevalence in individuals aged 20–54 years old [1], which is a demographic that is among the active users of social media.

Based on recent data, ~4 billion people worldwide use social media [2]. YouTube, one of the most widely used social media platforms for long-form video content, hosts a substantial number of videos related to migraines. However, the majority of these videos tend to focus on complementary and alternative medicine rather than providing educational content grounded in evidence-based research [3], TikTok is one of the newer, more popular, and fastest-growing social media platforms utilizing short-form videos accessible to the general public [4]. In the past five years, it has overtaken Facebook, Twitter, and Instagram as the most frequently downloaded application. With the evolving influence of social media, the younger generation have been more exposed to short-form, video-based platforms like TikTok, along with Instagram, Snapchat, or Facebook’s video reels. Studies in both in Asia and the United States have found that younger individuals tend to turn to these short-form video contents from TikTok for health information [3–5]. A growing number of articles have been evaluating TikTok videos as part of social media analyses, covering common medical conditions such as myocardial infarction, diabetes, osteoporosis, and psychiatric disorder [3, 4, 6–10]. These studies have found the information on TikTok to be inaccurate, raising concerns on the spread of misinformation among viewers, which can pose a threat to public health communication. In the local setting, a published study explored the theme of vaccine hesitancy in TikTok reported that video content on TikTok may influence vaccine-related opinions among Filipino audiences [11]. This further supports the role of TikTok as a widely used source of health information and underscores the potential public health implications of variable, inaccurate quality and reliability in common health conditions, particularly migraine.

In this study, we aimed to describe the available TikTok videos discussing migraine, and evaluate its content in terms of reliability, quality, and understandability. The study also aimed to investigate factors of these videos associated with increased engagement, likes, and shares, with the general intent to identify and address health misinformation about migraine in social media.

## Methods

### Search strategy

This research was a cross-sectional observational study conducted in July 2025. Data gathering was initiated by using newly created TikTok accounts with no prior interactions to avoid algorithmic personalization [4, 12, 13]. Location services were disabled on the cellphones used. The two independent raters (IMDJ and RDNB) did their search on the same date, within the same vicinity, at the same time to target a similar IP address, to ensure less variability in the data extracted [14]. Each rater viewed the included videos separately. The keyword ‘#migraine’ was used in the ‘Top’ search function on TikTok. The first 200 videos were the desired number for data collection [12, 13, 15]. However, in the search done by the two raters, the videos were limited to 137 for Rater 1 and 180 for Rater 2, respectively. These videos were extracted and details were placed on a spreadsheet, where they were screened based on the eligibility criteria set.

### Inclusion and exclusion criteria

Videos included had a runtime of five seconds to seven minutes, and were in English or Filipino. After compiling the initial total of 317 videos, 268 videos were excluded. Videos that did not last for at least 15 seconds, or had content pertaining to the song entitled ‘Migraine’, including slideshows, lyric videos, were not included in the analysis.

### Data extraction

All essential data were recorded using Google Sheets. Video details such as date posted, length of video (in seconds), number of views, likes, comments, and reposts were encoded. The videos were categorized based on their source and content using the following for source: (i) patient, (ii) healthcare provider, (iii) non-medical professional, (iv) healthcare institution, (v) university, (vi) non-government organization, (vii) others, including influencers, complementary and alternative medicine practitioners; and, the following categories were used for content: (a) epidemiology, (b) risk factors, (c) clinical presentation, (d) diagnosis, (e) management, and (f) personal experience. These were determined by the channel description and type of uploaded videos. Engagement metrics (likes, views, shares) were recorded as rounded counts as displayed (e.g. 1.2 K, 10 K, 1 M), since exact numbers are not visible in the TikTok interface [16]. Videos extracted were publicly available from the search and time of rating.

### Video evaluation

Two raters (IMDJ and RDNB) viewed and rated all of the screened videos independently using three scales: the Global Quality Scale (GQS), the modified version of DISCERN (mDISCERN), and the Patient Education Materials Assessment (PEMAT), which have all been frequently used in the evaluation of TikTok video quality on health information [3, 4, 6–9, 15, 17, 18]. The GQS is a five-point Likert scale which assesses the overall quality of a video based on its flow and usefulness to patients [4, 5]. The mDISCERN tool will be used to evaluate the reliability of each of the videos. This tool consists of five questions answerable by ‘Yes’ or ‘No’, for a total score of zero to five points. A score of zero indicates Low Reliability, while a score of five indicates High Reliability of the content of a video [12, 19]. Lastly, the PEMAT score is a 26-item tool evaluating the Understandability and Actionability of the content of an audiovisual material. Each item will be answered by ‘Agree’ or ‘Disagree’, which will be scored one or zero, respectively [20]. The higher the score, the more the content is interpreted to be understandable and/or actionable [20].

### Statistical analysis

Data were analyzed using JASP 0.19.0, a downloadable statistical analysis software from Wagenmakers. Inter-rater reliability for the scales were used using Spearman’s Rank Correlation, with an alpha of <0.05 used. This provides a direct assessment for two raters, as the appropriate statistic for non-parametric tests. Correlations between the video parameters and the scores for each tool will also be used as appropriate.

## Results

**Figure 1:**
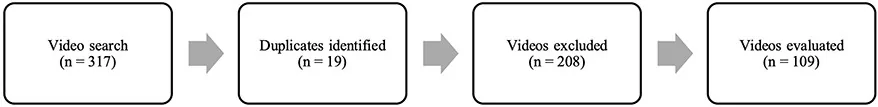
Video screening and extraction process.

After the initial search, a total of 317 videos were screened. Nineteen videos were identified as duplicates (6.4% overlap rate), excluding 208 videos, yielding a final sample of 109 unique videos. These videos were excluded due to content (*n* = 154), duration (*n* = 185), and language (*n* = 9). Specifically, a number of videos were removed from the final pool due to duration and content. Videos that did not last for at least 15 seconds, or had content pertaining to the song entitled ‘Migraine’, including slideshows, lyric videos, were not included in the analysis. A total of 109 videos remained for evaluation (see Fig. 1).

**Table 1:** Spearman’s correlations.

Variable		GQS	PEMATb TOTAL	PEMAT U	PEMAT A	Likes	Views	Comments	Shares	Length (sec)
**GQS**	Spearman’s rho	–								
	*P*-value	–								
**PEMAT TOTAL**	Spearman’s rho	**0.739**	**–**							
	*P*-value	**<.001**	**–**							
**PEMAT U**	Spearman’s rho	**0.803**	**0.953**	–						
	*P*-value	**<.001**	**<.001**	–						
**PEMAT A**	Spearman’s rho	0.309	**0.679**	**0.499**	–					
	*P*-value	.003	**<.001**	**<.001**	–					
**Likes**	Spearman’s rho	−0.026	**0.074**	0.015	**0.334**	–				
	*P*-value	.807	**<.001**	.892	**<.001**	–				
**Views**	Spearman’s rho	−0.072	**0.045**	−0.015	0.290	**0.947**	–			
	*P*-value	.502	**<.001**	.885	.006	**<.001**	–			
**Comments**	Spearman’s rho	0.088	0.183	0.135	**0.380**	**0.904**	**0.845**			
	*P*-value	.414	.087	.207	**<.001**	**<.001**	**<.001**			
**Shares**	Spearman’s rho	6.967 × 10^−4^	0.158	0.085	**0.406**	**0.944**	**0.936**	**0.894**		
	*P*-value	.995	.138	.431	**<.001**	**<.001**	**<.001**	**<.001**		
**Length (sec)**	Spearman’s rho	**0.393**	**0.420**	**0.391**	0.290	0.085	0.065	0.247	0.208	
	*P*-value	**<.001**	**<.001**	**<.001**	.006	.431	.545	.020	<.050	

GQS – Global Quality Scale; PEMAT – Patient Education Materials Assessment Tool; PEMAT U – Patient Education Materials Assessment Tool Understandability; PEMAT A – Patient Education Materials Assessment Tool Actionability.

The videos evaluated were uploaded from November 2021 to June 2025. Fifty-five percent of the videos were uploaded by influencers or sellers of migraine treatment merchandize (*n* = 60). Other uploaders categorized as physicians or physician groups comprised 32% of the videos (*n* = 35), followed by 6% each for non-MDs like chiropractors, pharmacists, or therapists (*n* = 7), and educational sites (*n* = 7).

The median length of the TikTok videos included was 53 seconds, with the longest uploaded by a physician group. Videos with the most views were those uploaded by physicians (median = 368 000 views), while videos with the most likes were those uploaded by influencers and physicians, particularly those talking about quick migraine hacks or home treatments (median = 11 650 likes).

The majority of the videos examined (56%, *n* = 61) talked about the management of migraines, followed by personal experience (40.4%, *n* = 44), and clinical presentation (31.2%, *n* = 34). Lesser videos discussed risk factors, diagnosis, and epidemiology.

Spearman’s rank correlation was used to calculate inter-rater reliability. This yielded a significant positive correlation for GQS (0.912; *P* < .001), PEMAT Understandability (0.839; *P* < .001), PEMAT Actionability (0.705; *P* < .001), and the overall PEMAT score (0.805; *P* < .001). However, a negative correlation was found for mDISCERN scores (0.121; *P* < .263).

Median combined scores for GQS and mDISCERN revealed low scores (median score = 0 to 1). The majority of videos had low quality (63%). Only two videos (1.8%) had high reliability (score of four to five), with no video scoring a 5.0. Similarly, only five videos (4.5%) had high quality (score of four to five), with only one video scoring a 5.0. Physicians, physician groups, and healthcare professionals who uploaded videos received higher scores on the GQS and mDISCERN for reliability and quality. The median scores for PEMAT understandability (~48%) and PEMAT actionability (~31%) also showed relatively low scores. There were 25 videos (23%) that had higher scores (>70%) and were deemed more understandable, and those in particular were uploaded by physicians and physician groups, highlighting the clinical presentation and management of migraines. Consequently, only ten videos (9%) were deemed actionable based on their scores. Overall, 14 videos passed the 70% mark for PEMAT total scores.

On analysis, higher scores overall in the PEMAT showed a positive correlation with higher scores on the GQS [PEMAT Total *r* = 0.739 (*P* < .001); with GQS *r* = 0.803 (*P* < .001)]. The number of likes had a positive correlation with higher PEMAT actionability scores [Likes *r* = 0.334 (*P* < .001)], but not understandability; overall, with positive correlation with PEMAT total scores [Likes *r* = 0.074 (*P* < .001); with PEMAT total *r* = 0.803 (*P* < .001)]. The number of views had a positive correlation with PEMAT Total [View *r* = 0.045 (*P* < .001)], but in contrast, a negative correlation in PEMAT actionability. An increased number of likes was correlated with an increased number of views [Views with Likes *r* = 0.947 (*P* < .001)]. Videos with higher likes, comments, and shares, had a positive correlation with PEMAT actionability scores [Likes with PEMAT A *r* = 0.334 (*P* < .001); Comments with PEMAT A *r* = 0.380 (*P* < .001); Shares with PEMAT A *r* = 0.406 (*P* < .001)]. Length of the video (in seconds) had a positive correlation with GQS and PEMAT total scores [Length with GQS *r* = 0.393 (*P* < .001); Length with PEMAT Total *r* = 0.420 (*P* < .001)], but a negative correlation in likes, views, comments, and shares (see Table 1).

## Discussion

Due to the popularity of social media, evaluating the health information they present has been evaluated in the past involving YouTube content [4, 10–12, 21–23]. There are few studies that have evaluated TikTok videos [3–5, 13, 15], with one locally published study [11]. To our knowledge, this is the only study to evaluate the quality of TikTok videos as a source of information on migraine in our low-to-middle income setting.

Most of the videos included in this study were uploaded by influencers and sellers, which had lower quality as scored. Better quality was seen in videos uploaded by the physician group, which would tend to be longer videos. However, these videos had lower community interactions such as likes, shares, and comments. Similar findings were seen in studies evaluating YouTube content, where shorter, popular videos of lesser quality topped longer, more factual, accurate videos of higher quality [13, 23].

Interestingly, shorter videos seemed to score better in the actionability component of the PEMAT, increasing its overall total. These videos discussed migraine treatment or ‘hacks’ uploaded by influencers demonstrating how to make use of this. Higher scores on the actionability may be due to these video types, since the uploader would give specific instructions on how to make use of a certain recommendation. A number of the videos evaluated were laypeople narrating their personal experiences of migraine, what they felt, and how they managed it. This may be due to social media encouraging individuals to share their own stories, providing an avenue to seek support or some advice in the comments section [24]. This sense of community and belonging simulated online elevates their quality of life and gives them hope in relation to their disease [6, 9, 24], and has had growing significance since the pandemic. Additionally, narrations of first hand experiences with migraine resonate more to the audience, generating more empathy in the comments and viewers [6]. Videos uploaded by physicians and other healthcare professionals indeed showed better quality when compared to the others [4, 12, 13, 21, 22]. We found that some may not always be reliable due to a lack of proper citation of references and disclosure of bias. The increasing popularity of TikTok is also related to these short-form, short-duration types of videos [3, 4, 10, 13, 15, 16]. Hence, to be able to adhere to the short-form video format, some uploaders may have to cut essential information, leading to poorer quality videos. Although these findings highlight the potential for increased health misinformation online, the World Health Organization (WHO) has identified digital platforms as important channels for public health communication to reach target populations. With the use of this content, especially in low-to-middle income countries where resources and accessibility can be limited, healthcare professionals and their institutions are encouraged to create their own content for awareness and more accurate health information [25]. Strategic use of social media by these institutions may help mitigate the misinformation and eventually improve health literacy.

## Limitations

The authors acknowledge several limitations that have been present in this study. One limitation noted was the language used, only limiting videos in English and Filipino. This was done to be applicable to the setting of the study. The number of videos excluded due to content was also significant. The search was only limited to the videos using the #migraine hashtag to simulate users doing an actual search, without additional specifiers such specific to treatment, diagnosis, and reliable healthcare. Moreover, algorithmic sampling bias may have been present, as TikTok’s ‘Top’ search results are influenced user engagement. Despite measures to standardize the search process, the extracted videos may reflect content promoted by TikTok, than all the migraine-related content on the platform, which may limit the generalizability of the findings. Additionally, the dynamic nature of TikTok’s algorithm makes it difficult to control which videos are displayed. Because the analyzed videos were uploaded over a nearly four-year period, changes in TikTok’s platform features, health-content policies, and user engagement patterns may have affected the characteristics of the sampled videos. Future studies should consider temporal analyses in social media content over time.

Although the PEMAT, GQS, and mDISCERN are validated assessment tools, inter-rater reliability was acceptable only for the PEMAT and GQS, but not for the mDISCERN. As such, the mDISCERN findings should be interpreted with caution. Some degree of subjectivity may remain, as both reviewers had healthcare backgrounds that may have influenced their assessment of health information quality. Lastly, the PEMAT was not specifically designed for short-form social media content. While its domains of understandability and actionability are relevant to health-related videos, certain aspects of TikTok’s format may not be fully captured by the tool, which could have influenced the assessment of some videos.

## Conclusion

The majority of TikTok videos on migraine were found to be of low quality, reliability, and understandability, predominantly uploaded by non-healthcare professionals. These findings highlight the potential role of social media platforms such as TikTok in contributing to the lack of health literacy by its audiences. Viewers should be cautioned to evaluate the source of migraine-related content and prioritize information created by qualified healthcare professionals. Moreover, this study emphasizes the need for collaborative efforts to make social media a proper medium for health education. Future research could evaluate the impact of healthcare institution-led social media initiatives on the dissemination of accurate health information on migraine.

## Data Availability

The data underlying this article will be shared on reasonable request to the corresponding author.
